# Gene and pathway based burden analyses in familial lymphoid cancer cases: Rare variants in immune pathway genes

**DOI:** 10.1371/journal.pone.0287602

**Published:** 2023-06-28

**Authors:** Sneha Ralli, Samantha J. Jones, Stephen Leach, Henry T. Lynch, Angela R. Brooks-Wilson

**Affiliations:** 1 Department of Biomedical Physiology and Kinesiology, Simon Fraser University, Burnaby, British Columbia, Canada; 2 Canada’s Michael Smith Genome Sciences Centre, BC Cancer, Vancouver, British Columbia, Canada; 3 Hereditary Cancer Center, Creighton University, Omaha, Nebraska, United States of America; CNR, ITALY

## Abstract

Genome-wide association studies have revealed common genetic variants with small effect sizes associated with diverse lymphoid cancers. Family studies have uncovered rare variants with high effect sizes. However, these variants explain only a portion of the heritability of these cancers. Some of the missing heritability may be attributable to rare variants with small effect sizes. We aim to identify rare germline variants associated with familial lymphoid cancers using exome sequencing. One case per family was selected from 39 lymphoid cancer families based on early onset of disease or rarity of subtype. Control data was from Non-Finnish Europeans in gnomAD exomes (N = 56,885) or ExAC (N = 33,370). Gene and pathway-based burden tests for rare variants were performed using TRAPD. Five putatively pathogenic germline variants were found in four genes: *INTU*, *PEX7*, *EHHADH*, and *ASXL1*. Pathway-based association tests identified the innate and adaptive immune systems, peroxisomal pathway and olfactory receptor pathway as associated with lymphoid cancers in familial cases. Our results suggest that rare inherited defects in the genes involved in immune system and peroxisomal pathway may predispose individuals to lymphoid cancers.

## Introduction

Lymphoid cancers arise at various stages of B- and T-cell differentiation. There are more than 80 subtypes of lymphoid cancer [1] that can be grouped into three types: Hodgkin lymphoma, non-Hodgkin lymphoma, and other lymphoproliferative disorders [2]. Non Hodgkin lymphoid neoplasms are the 7th most common cancer in North America [3]. Lymphoid cancer types and subtypes represent heterogeneous group of diseases with different genetic mechanisms, clinical presentation and histological appearance and ages of onset [4, 5]. Lymphoid cancers are complex disorders caused by various genetic [6, 7] and environmental factors [8, 9].

Genetic factors for lymphoid cancers include common and rare variants [10]. Familial studies mostly detect rare variants with high effect sizes. Family-based sequencing studies have identified rare lymphoid cancer predisposition variants in several genes, including *KDR* [11], *KLHDC8B* [12], *POT1* [13], *ACAN* [14], *NPAT* [15], and others [7]. In addition, significant linkage has been found for chronic lymphocytic leukemia (CLL), at 2q21.2, which contains the chemokine receptor (*CXCR4*) gene [16].

In contrast, genome wide association studies (GWAS) of cases and controls often identifies common variants with smaller effect sizes. Susceptibility genes that are involved in B-cell development, immune response [17], DNA repair [18] and immune function [19] have been associated with various subtypes of lymphoid cancers. The NHGRI-EBI GWAS catalog [20] contained 654 associations for lymphoid cancers as of October 3, 2022 with the most significant association being for B-cell acute lymphoblastic leukemia with a variant rs10821936-C (*p-value*: *1X10*^*-106*^) in *ARID5B* [21]. The heritability estimate for Hodgkin lymphoma was 28.4% in a Swedish population study [22]. The heritability of non-Hodgkin lymphoma varies by subtype; for instance, for CLL it is 16–34% [17, 23] and for DLBCL it is 16% [24]. Although many susceptibility loci have been identified for lymphoid cancers, a substantial fraction of the disease heritability remains unexplained. Some of this ‘missing heritability’ could be due to rare variants with small effect sizes [25–27].

Rare variant association studies (RVAS) are used to identify low allele frequency variants with small effect sizes. In RVAS, the number of cases with rare damaging variants in each gene or group of genes (e.g., pathway) is compared to that of controls to generate one statistical test result per gene or group of genes, instead of one per variant. Various statistical tests have been developed to detect rare variant associations such as the burden test, the adaptive burden test, the variance-component test, and the omnibus test, all of which have been reviewed previously [28, 29]. Study designs and considerations for performing RVAS have also been described [29–31]. RVAS has been applied in lymphoid cancer cases with a mature B-cell non-Hodgkin lymphoma subtype, and identified a burden of rare germline variants in *CHMP6* and *GSTA4* [32]. Unlike GWAS, where the genome-wide significance threshold is defined at p = 5×10^8^, there is no standard threshold for RVAS, as the number of tests performed depends on the sequencing platform used, the depth of coverage, and how variants are aggregated in the test [33].

To improve our understanding of the etiology of lymphoid cancers, we performed gene- and pathway-based RVAS to test for an aggregated burden of rare, deleterious variants in gene(s) and pathway(s), in familial lymphoid cancer cases compared to controls from public databases. Test Rare vAriants with Public Data (TRAPD) was used [34], as it allows for the use of controls from public databases such as the large exome sequence databases Genome Aggregation Database (gnomAD) [35] and ExAC [36].

## Methods

### Participants

The study was approved by the Research Ethics Board of BC Cancer and the University of British Columbia with Simon Fraser University as the harmonized partner board. All participants provided written informed consent.

Cases were derived from the Lymphoid Cancer Families Study (LCFS) [37]. Eligible families had two or more members, living or deceased, with a lymphoid cancer. The type and subtype of the cancers were classified according to the hierarchical classification provided by the International Lymphoma Epidemiology Consortium (InterLymph) Pathology Working Group [2]. Families (N = 39) were prioritized for exome sequencing based on the number of family members affected by lymphoid cancer and the number of germline samples available for sequencing.

### Whole exome sequencing

Samples from 39 lymphoid cancer families, including 39 early onset cases and relatives, were part of a batch of 92 samples subject to whole exome sequencing, performed using an Illumina HiSeq2500 instrument in High Output mode with HCS (v2.2.68) at the Genome Sciences Centre (Vancouver, British Columbia, Canada). DNA extraction for 37 early onset cases was from blood; for two cases, saliva samples were used. Briefly, library preparation involved shearing of DNA using Covaris E-Series (Covaris, E210) to an average fragment size of 250 bp and using NEB Paired-End Sample Prep Kit (NEB, E6000B-25B; protocol as per kit version 1.1) for phosphorylation, d-A tailing, adaptor ligation, and PCR enrichment of sheared samples. Capture was performed with Agilent SureSelect v5 + UTR kit capture probes using four libraries per capture, for a total of 23 captures. The protocol was carried out according to the Agilent SureSelect Target Enrichment System for Illumina Multiplexed Sequencing. Amplification was performed using the HiSeq PE Cluster Kit v4 (Illumina, PE-401-4001) and cBot Cluster Station. Sequencing was performed using the HiSeq SBS Kit v4 (Illumina, FC-401-4003) and the Illumina HiSeq2500 System, generating 125 bp reads.

### Variant calling

Reads were mapped and variants called at the Bioinformatics core facility at the Genome Sciences Centre. Paired-end reads were mapped against the human reference genome (GrCh37) using the Burrows-Wheeler Aligner (version 0.5.7) [38]. Aligned reads were filtered and sorted using Sambamba (version 0.5.52) [39] and the BAM file was generated using GATK’s (version 4.0.2.1) HaplotypeCaller [40] with the–L parameter to restrict the analysis to exome specific intervals. Variants were jointly called to produce genotypes for all variant positions in all samples, according to GATK’s best practice recommendations [41] to generate a single raw VCF file with 290,549 variants.

### Variant quality filtering

**Fig 1:**
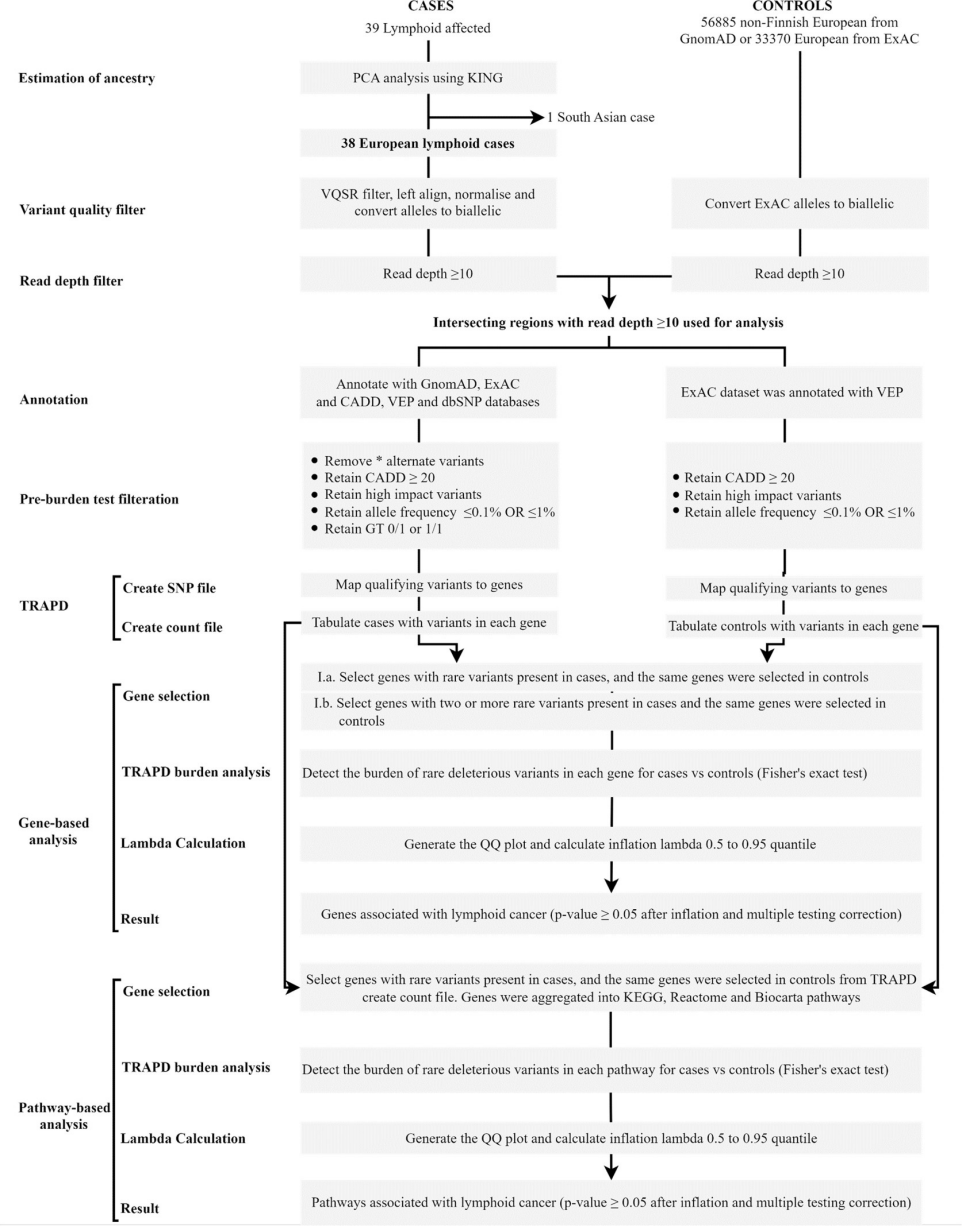
Study workflow.

The raw VCF file containing single nucleotide variants (SNV) and insertions and deletions (indels) was filtered for variant quality using Variant Quality Score Recalibration (VQSR) with truth sensitivity at 99.0% for both SNVs and indels. Variants that did not pass VQSR were removed. VQSR filtering was performed on a batch of 92 exomes for multiple projects including this study. A subset of VCF file containing only the genotype data for the early onset 39 cases was generated using bcftools (version 1.13) [42]. Workflow for the rare variant association study is summarized in Fig 1. Multiallelic sites were converted into biallelic sites with left-alignment, and normalization of variants were performed by using Bcftools [42]. Sequencing, variant calling, and filtering methods for control set GnomAD and ExAC were previously described [35, 36]. Variants that had a PASS filter status in both cases and controls were retained for RVAS.

### Estimation of ancestry

Input.bed files for ancestry estimation were generated using PLINK (version 1.9) [43, 44]. A principal component analysis (PCA) was conducted using KING (version 2.2.7) [45] with the 1000 Genomes Project dataset as reference. PCA plots were produced using the command—rplot with the e1071 package in R version 3.6.0 (S1 Fig). For gnomAD and ExAC controls, the ancestries were as described [35, 36].

### Read depth filtering

Different exon capture methods were used for cases and controls. To reduce potential bias due to differences in coverage, the proportion of individuals covered at a read depth of > 10x was calculated separately for cases and controls. Bases with at least 90% of the samples covered at >10x in both cases and controls were retained for analysis. The coverage files for gnomAD exome and ExAC controls were obtained from the gnomAD consortium [46].

### Annotation

Annotation was performed using Snpsift (version 5.0) [47] and Variant Effect Predictor (VEP) (version 103) [48] using the Genome Aggregation Database (gnomAD 2.2.1) [35], ExAC [36], and Combined Annotation Dependent Depletion (CADD) (version 1.6) [49]. European allele frequencies were annotated from gnomAD and ExAC databases. Variants not annotated for allele frequency in these databases were annotated manually with the dbSNPs Allele Frequency Aggregator (ALFA) European allele frequency.

### Pre-burden test filtration

Variants in cases and controls were filtered to retain only those with CADD Phred score ≥ 20 and high impact variants such as splice acceptor, splice donor, stop-gain, frameshift, stop lost, start lost from VEP (version 103). Analyses were limited to variants with allele frequency ≤ 1% (rare variants) or ≤ 0.1% (very rare variants). Variant data from cases was filtered to retain only those genotypes where at least one case had an alternate allele.

### Burden tests

Burden testing was performed to identify genes and pathways with a higher frequency of rare deleterious variants in cases compared to publicly available European controls from gnomAD (N = 56,885) and ExAC (N = 33,370) using the Test Rare vAriants with Public Data (TRAPD) method. TRAPD utilizes summary statistics for the variants from publicly available control data and allows for the application of both dominant and recessive models. TRAPD generates three files: a SNP file with qualifying rare variants mapped to genes, a count file with tabulated count of rare variants in the genes, and a burden file with p-value for each gene and pathway using a two-sided Fisher’s exact test.

Gene-based analysis included only genes that, after filtering, had one or more variants in at least one case. Therefore, for gene-based burden analysis, the same genes with rare damaging variants present in cases were selected from the controls using the TRAPD count file before carrying out the burden tests. In addition, a subset of these genes was chosen to include genes with two or more variant counts in cases before carrying out burden tests.

Pathway-based burden analysis was performed by aggregating genes into pathways based on three widely-used databases: Reactome [50], KEGG [51], and Biocarta. The molecular signatures database from the Broad institute [52] (http://www.broadinstitute.org/gsea/downloads.jsp) was used to download KEGG and Biocarta pathways. Reactome was downloaded from https://reactome.org/download-data. The Reactome database has low-level and high-level pathway hierarchies; here we used the low-level pathways. Genes with rare variants present in cases were selected from the controls using the TRAPD count file and grouped into pathways. The variant counts were summed to obtain a per-pathway rare variant count for pathway-based burden analysis.

Burden analysis was performed for the dominant model. The p-values generated by TRAPD were corrected for the inflation factor, a measure of population stratification. To measure the inflation factor, lambda 0.5 to 0.95 was calculated which fit a line to the points within 0.5 and 0.95 quantiles, as described previously [53]. Quantile-Quantile (QQ) plots were constructed using R (v3.6.0). Multiple testing corrections were performed based on the number of genes analysed at allele frequencies ≤1% and ≤0.1%. Genes or pathways were considered to be associated with lymphoid cancer if the p-value after correction for the inflation factor and multiple testing was ≤ 0.05. For the genes and pathways with significant associations, variants were visually inspected using the Integrative Genomics Viewer (version 2.4) [54] and excluded if deemed an artifact. Variants are deemed as artifacts if they show strand bias or allele frequency inconsistent with the heterozygous variant, have low base-quality of the alternate allele in the reads or low mapping quality.

## Results

Exome sequencing included an early onset case from each lymphoid cancer family (N = 39). The types/subtypes of the 39 cases are shown in Table 1. Estimation of ethnicity resulted in 38 cases of European ancestry that were included in rare variant burden analyses, and one case of South Asian ancestry (S1 Fig) that was excluded from further analysis.

**Table 1:** Lymphoid cancer families included in exome sequencing and cases chosen for RVAS.

Family ID	Number of family members with a lymphoid cancer	Chosen Case			
		Age of onset	Sex	Lymphoid cancer type	Lymphoid cancer subtype
10	2	24	Female	Hodgkin lymphoma	Nodular sclerosis
12^+^	3	35	Male	Hodgkin lymphoma	Nodular sclerosis
14	4	52	Female	non-Hodgkin lymphoma	Mucosa-associated lymphoid tissue lymphoma
19	4	29	Male	Hodgkin lymphoma	Nodular sclerosis
32	2	34	Male	non-Hodgkin lymphoma	Chronic lymphocytic leukemia
54	3	48	Female	Non-Hodgkin lymphoma	Follicular lymphoma
59	3	73	Male	Non-Hodgkin lymphoma	Lymphoplasmacytic lymphoma
66	3	41	Male	Non-Hodgkin lymphoma	Follicular lymphoma
68	2	46	Male	Non-Hodgkin lymphoma	Follicular lymphoma
69	3	46	Male	Non-Hodgkin lymphoma	Follicular lymphoma
71	3	50	Male	Non-Hodgkin lymphoma	Diffuse large B-cell lymphoma
76	4	30	Female	Hodgkin lymphoma	Nodular sclerosis
89	4	52	Male	Non-Hodgkin lymphoma	Chronic lymphocytic leukemia
100	2	62	Male	Non-Hodgkin lymphoma	Chronic lymphocytic leukemia
108	2	75	Female	Non-Hodgkin lymphoma	Chronic lymphocytic leukemia
110	2	67	Female	Non-Hodgkin lymphoma	Chronic lymphocytic leukemia
126	3	35	Female	Hodgkin lymphoma	Nodular sclerosis
129	2	9	Male	Non-Hodgkin lymphoma	Burkitt lymphoma
133^+^	5	19	Male	Hodgkin lymphoma	Nodular lymphocyte predominant Hodgkin lymphoma
134	2	34	Male	Non-Hodgkin lymphoma	Diffuse large B-cell lymphoma
154	6	58	Female	Lymphoid neoplasm, not other specified	Lymphoid neoplasm, not otherwise specified
157	2	58	Male	Non-Hodgkin lymphoma	Chronic lymphocytic leukemia
162	2	48	Female	Non-Hodgkin lymphoma	Chronic lymphocytic leukemia
173	3	29	Female	Hodgkin lymphoma	Nodular sclerosis
175*	2	40	Male	Non-Hodgkin lymphoma	Diffuse large B-cell lymphoma
189	5	57	Female	Non-Hodgkin lymphoma	Follicular lymphoma
195	2	46	Male	Non-Hodgkin lymphoma	Diffuse large B-cell lymphoma
197	3	47	Male	Non-Hodgkin lymphoma	Follicular lymphoma
213	2	21	Female	Hodgkin lymphoma	Nodular sclerosis
226	3	49	Female	Non-Hodgkin lymphoma	Follicular lymphoma
229	2	45	Male	Non-Hodgkin lymphoma	Chronic lymphocytic leukemia
238	4	78	Female	Non-Hodgkin lymphoma	Follicular lymphoma
255	2	27	Male	Non-Hodgkin lymphoma	Primary mediastinal large B-cell lymphoma
1705	8	36	Female	Lymphoid neoplasm, not other specified	Lymphoid neoplasm, not other specified
4541	3	22	Male	Hodgkin lymphoma	Hodgkin Lymphoma, not other specified
35	2	50	Female	Non-Hodgkin lymphoma	Chronic lymphocytic leukemia
113	3	65	Female	Non-Hodgkin lymphoma	Plasma cell myeloma
204	2	58	Male	Non-Hodgkin lymphoma	Small lymphocytic lymphoma
3474	4	68	Female	Lymphoid neoplasm, not other specified	Lymphoid neoplasm, not other specified

* Excluded after ethnicity analysis.

^+^ DNA sample was extracted from Saliva.

ExAC (N = 33,370) and GnomAD exomes (N = 56,885) with Non-Finnish European ethnicity were used as controls. Read depth filtering to exclude any bases that did not have read depth 10 or more, in at least 90% of cases and 90% of a control set, resulted in slightly different sets of variants being included in the analyses with GnomAD controls vs. those with ExAC controls. After read depth filtering using gnomAD controls, 102,385 variants remained, whereas 103,427 variants remained when controls were from ExAC. After filtering to retain high impact rare deleterious variants using an allele frequency cut-off of ≤1%, there were 467 rare variants with GnomAD as controls, and 400 rare variants with ExAC as controls, whereas at an allele frequency cut-off of ≤0.1%, there were 294 rare variants with GnomAD as controls and 276 rare variants with ExAC as controls.

### Gene-based burden analysis

Burden tests were performed at the gene and pathway levels to identify if there was a higher burden of rare variants in lymphoid cancer cases than in controls from GnomAD exomes and ExAC. First, we analyzed genes with one or more variant counts in cases. There were 404 and 258 genes containing deleterious variants with allele frequencies ≤1% and ≤0.1% analysed when the controls were GnomAD exomes, and 355 and 210 genes at allele frequencies ≤1% and ≤0.1% when the control set was ExAC. There were no significant associations between lymphoid cancer and any individual gene, with either control set or at either allele frequency, after correcting for inflation and for multiple testing. The inflation factors for gene-based analysis for both the controls and allele frequency are summarised in S1 Table.

We then limited analysis to genes with two or more variant counts present in any of the 39 cases. At allele frequency ≤1%, 43 and 39 genes were analysed when the controls were GnomAD exomes and ExAC, respectively. Statistical power for the cases and controls when two or more variants were analysed using genpwr [55] are summarized in S2 Table. Lymphoid cancer associated genes and variants are summarized in Table 2. *INTU* was the only gene associated with lymphoid cancer after multiple testing correction for 43 genes with GnomAD exomes and 39 genes with ExAC controls. For allele frequency ≤0.1%, 7 and 5 genes were analysed when the controls were GnomAD exomes and ExAC, respectively. Four genes, *INTU* (p-value 0.005), *ASXL1* (p-value 0.009), *EHHADH (*p-value 0.03) and *PEX7* (p-value 0.02), were associated with lymphoid cancer after multiple testing correction for 7 genes when GnomAD exomes were used as controls. Three genes, *INTU* (p-value 0.004), *PEX7* (p-value 0.02), *and EHHADH* (p-value 0.02) were associated with lymphoid cancers after multiple testing correction for 6 genes with ExAC controls. Data underlying the results of burden testing are summarized in S3 Table.

**Table 2:** Five rare variants in four genes significantly associated with lymphoid cancers in genes with two or more variant counts.

Family ID	rsid	Gene	HGVS	AF^1^	Conse-quence	Allele frequency ≤1%		Allele frequency ≤0.1%		Case			Family members			
						GnomAD controls p-value*	ExAC controls p-value*	GnomAD controls p-value*	ExAC controls p-value*	Diag-nosis	Age of onset	Sex	Number of other family members exome seq’d	Relation-ship	Diag-nosis	Carries variant?
32	rs781090244	*EHHADH*	NM_001966.4:c.594_595del	0.00013	Splice Acceptor			0.03	0.02	CLL	34	Male	1	Sister	FOLL	No
134										DLBCL	34	Male	1	Mother	CLL	No
129	rs1347837845	*INTU*	NM_015693.4:c.466C>T	0	Stop-gain	0.04	0.05	0.005	0.004	BL	9	Male	1	Sister	NS	No
100	rs1450337930	*INTU*	NM_015693.4:c.581G>A	0	Stop-gain					CLL	62	Male	1	Mother	CLL	Yes
76	rs1805137	*PEX7*	NM_000288.4:c.875T>A	0.0007	Stop-gain			0.02	0.02	NS	30	Female	3	Mother	Skin (BCC)	Yes
														Father	LPL	No
														Maternal cousin	NMZL	No
129										BL	9	Male	1	Sister	NS	Yes
59	rs750318549	*ASXL1*	NM_015338.6:c.1934dup	0.0005	Frame-shift			0.009		LPL	73	Male	1	Sister	CLL	No
35										CLL	50	Female	0	--	--	No

HGVS = Human Genome Variation Society. CLL = chronic lymphocytic leukemia, BL = Burkitt lymphoma, NS = Nodular Sclerosis, DLBCL = diffuse large B-cell lymphoma, LPL = Lymphoplasmacytic lymphoma, BCC = Basal cell carcinoma, NMZL = Nodal marginal zone lymphoma, FOLL = Follicular lymphoid cancer.

^1^ allele frequency from gnomAD, ExAC, 1000genomes or ALFA databases

* p-values shown here are after correcting for the inflation factor and multiple testing, when necessary

#### Examination of variants of interest in family members

For cases with rare variants in genes associated with lymphoid cancer, we looked at whether any affected relatives also exome sequenced carried the variant or not. *INTU* has two stop-gain variants of interest: NM_015693.4:c.466C>T (rs1347837845) and NM_015693.4:c.581G>A (rs1450337930), these novel variants were not reported in Clinvar. NM_015693.4:c.466C>T was found in a case from family 129 with Burkitt lymphoma at the age of 9. This participant had one family member (a sister) diagnosed with nodular sclerosis, not otherwise specified, at age 34; the sister does not carry the NM_015693.4:c.466C>T variant. Given that the sibling does not share the NM_015693.4:c.466C>T variant in *INTU*, it is likely that additional genetic factors might contribute to familial lymphoid cancer in this family. NM_015693.4:c.581G>A was observed in an individual with CLL at the age of 62 from family 100; his/her mother, who was diagnosed with CLL at the age of 71, also carried this variant. As both the parent and offspring with the same lymphoma subtype carry the NM_015693.4:c.581G>A variant, it is plausible that this variant could be involved in susceptibility to lymphoid cancer in this family.

A stop-gain variant NM_000288.4:c.875T>A (rs1805137) in *PEX7*, which is indicated in Clinvar as pathogenic, was observed in two cases from different families; one case had nodular sclerosis at age 30, while the other had Burkitt lymphoma at age 9. The case with nodular sclerosis had two additional family members affected with lymphoid cancer; a father and a maternal cousin, as well as a mother without lymphoid cancer but with basal cell carcinoma (BCC), who were exome sequenced. The father developed lymphoplasmacytic lymphoma at 61 years, and the maternal cousin developed nodal marginal zone lymphoid cancer at 28 years; they did not carry the NM_000288.4:c.875T>A variant. The mother, who developed BCC at 45, carried the variant. Given that the mother who is not affected with lymphoid cancer had the variant in *PEX7*, but the affected father and the cousin did not, the NM_000288.4:c.875T>A variant seems unlikely to contribute to lymphoid cancer in this family. The case with Burkitt lymphoma had one additional exome sequenced family member, a sister diagnosed with nodular sclerosis at the age of 34. She carries the NM_000288.4:c.875T>A variant. The case who developed Burkitt lymphoma had variation in both the *PEX7* and *INTU* and developed cancer at the age of 9. The shared NM_000288.4:c.875T>A variant amongst the siblings implies that the gene might contribute to the susceptibility of lymphoid cancer in this family.

A frameshift variant, NM_015338.6:c.1934dup (rs750318549) in *ASXL1*, was observed in two cases, one with lymphoplasmacytic lymphoma at the age of 73 and one with CLL at the age of 50. This variant is also reported in Clinvar as pathogenic/likely pathogenic. The case with lymphoplasmacytic lymphoma had a sister sequenced who developed CLL at the age of 78 but did not carry the NM_015338.6:c.1934dup variant. For the case with CLL, no additional family members were sequenced.

A splice acceptor deletion NM_001966.4:c.594_595del (rs781090244) in *EHHADH* was observed in two unrelated familial lymphoid cancer cases, one diagnosed with CLL at the age of 34 and one with DLBCL at the age of 34. This variant has unknown significance in Clinvar. For the case with DLBCL, a family member (mother) was exome sequenced who developed CLL at 72 and did not carry the NM_001966.4:c.594_595del variant. The other case with CLL also had a family member (sister) who was exome sequenced. She developed Follicular Lymphoma at 44 and did not carry the NM_001966.4:c.594_595del variant. Since affected family members of both cases did not have the same variant in *ASXL1* or *EHHADH*, it is plausible that other factors contribute to familial lymphoid cancer in these families.

### Pathway-based burden analysis

We obtained 1761, 186, and 292 pathways from the Reactome, KEGG, and Biocarta databases, respectively. Gene and variant counts generated in the gene-based burden analysis were collapsed into pathways. The variant allele frequency thresholds of ≤1% and ≤0.1% were applied while aggregating the variant counts and genes to pathways. P-values were corrected for genomic inflation when necessary. The inflation factors for pathway-based analysis for both the controls and allele frequency are summarised in S1 Table. For variants with an allele frequency of ≤1% with GnomAD exomes as controls, a total of 483 pathways were identified: 333 Reactome, 106 KEGG, and 44 Biocarta pathways. Twelve pathways (10 Reactome and 2 KEGG) showed significant association after corrections (Table 3). With ExAC controls, 308 Reactome, 94 KEGG, and 34 Biocarta pathways were detected, for a total of 436 pathways. Two KEGG pathways were significantly associated with lymphoid cancer after corrections (Table 3).

**Table 3:** Pathway-based burden test for variants with allele frequency ≤1% and ≤0.1%, with GnomAD exomes and ExAC as controls.

Pathway database	Pathways	Allele frequency ≤1%						Allele frequency ≤0.1%					
		Variant count in cases	Variant count in Controls (GnomAD)	P-value*	Variant count in cases	Variant count in Controls (ExAC)	P-value*	Variant count in cases	Variant count in Controls (GnomAD)	P-value*	Variant count in cases	Variant count in Controls (ExAC)	P-value*
KEGG	Olfactory transduction	15	4772	0.0001	14	2564	0.0003	6	349	8.9E-05	6	283	0.0004
KEGG	Peroxisome	6	609	0.0026	6	316	0.001	6	609	0.0022	6	315	0.0007
KEGG	Purine metabolism							4	274	0.02	4	151	0.01
Reactome	Post-translational protein phosphorylation							3	116	0.05	3	71	0.04
Reactome	Regulation of Insulin-like Growth Factor (IGF) transport and uptake by Insulin-like Growth Factor Binding Proteins (IGFBPs)							3	116	0.05	3	71	0.04
Reactome	FCERI mediated Ca+2 mobilization	4	90	0.0004				4	90	0.0003			
Reactome	FCERI mediated MAPK activation	4	90	0.0004				4	90	0.0003			
Reactome	Antigen activates B Cell Receptor (BCR) leading to generation of second messengers	4	110	0.0008				4	110	0.0007			
Reactome	FCGR activation	3	77	0.0173				3	77	0.01			
Reactome	FCGR3A-mediated IL10 synthesis	3	77	0.0173				3	77	0.01			
Reactome	FCGR3A-mediated phagocytosis	3	77	0.0173				3	77	0.01			
Reactome	Regulation of actin dynamics for phagocytic cup formation	3	77	0.0173				3	77	0.01			
Reactome	Role of LAT2/NTAL/LAB on calcium mobilization	3	77	0.0173				3	77	0.01			
Reactome	Role of phospholipids in phagocytosis	3	77	0.0173				3	77	0.01			
Reactome	DAP12 signaling	2	15	0.0483				2	15	0.04			
KEGG	Tryptophan metabolism							4	266	0.02			
KEGG	Retinol metabolism							4	275	0.02			
KEGG	Drug metabolism cytochrome p450							4	310	0.04			

To test for associations between lymphoid cancers and rare variants with allele frequency ≤0.1%, 346 pathways were analyzed with GnomAD exomes as controls. These included 231 Reactome, 90 KEGG, and 25 Biocarta pathways. Eighteen pathways (12 Reactome and 6 KEGG) showed a significant association with lymphoid cancer (Table 3). There were 284 pathways, with 197 from the Reactome database, 71 from KEGG, and 16 from Biocarta, when the controls were from ExAC. Five pathways showed significant association with lymphoid cancer, of which four were from Reactome and three were from KEGG (Table 3).

To identify which genes in the lymphoid cancer associated pathways had rare variants, we summarized the count of rare variants aggregated by genes in cases and controls (Table 4). The 18 pathways (6 from KEGG and 12 from Reactome) associated with lymphoid cancers included 34 genes that had rare variant counts. One of the pathways associated with lymphoid cancer is the antigen activated B cell receptor, which was significant with a corrected p-value for multiple testing and inflation factor of 0.0008 and 0.0007 for allele frequency ≤0.1% and ≤1% respectively, with gnomAD controls. This pathway was then collapsed into high-level Reactome pathway hierarchies for the adaptive immune system. The remaining 11 pathways from Reactome were grouped into three high-level pathways. One of these high-level pathways is for the innate immune systems comprising seven low-level hierarchy pathways. Five genes with rare variants, *SYK*, *IGLV3-25*, *IGLV7-43*, *BLK*, and *GRAP2*, are included in these adaptive and innate immune system pathways. The five variants, one in each gene, are present in 5 different lymphoid cancer cases. The pathways of olfactory transduction and peroxisome from KEGG were also associated with the lymphoid cancer across both the allele frequencies and control sets. Data underlying these findings are summarized in S4 Table.

**Table 4:** Genes and variant counts for pathways significantly associated with familial lymphoid cancer cases.

Genes	GnomAD controls				ExAC controls				Associated Pathways	Higher level hierarchy	Database
	AF ≤1%		AF ≤0.1%		AF ≤1%		AF ≤0.1%				
	Cases	Controls	Cases	Controls	Cases	Controls	Cases	Controls			
*BLK*	1	33	1	33					Antigen activates B Cell Receptor (BCR) leading to generation of second messengers	Adaptive Immune System	Reactome
*IGLV3-25*	1	27	1	27							
*IGLV7-43*	1	48	1	48							
*SYK*	1	2	1	2							
*GRAP2*	1	13	1	13					DAP12 signaling	Innate Immune System	
*SYK*	1	2	1	2							
*GRAP2*	1	13	1	13					FCERI mediated Ca+2 mobilization		
*IGLV3-25*	1	27	1	27							
*IGLV7-43*	1	48	1	48							
*SYK*	1	2	1	2							
*GRAP2*	1	13	1	13					FCERI mediated MAPK activation		
*IGLV3-25*	1	27	1	27							
*IGLV7-43*	1	48	1	48							
*SYK*	1	2	1	2							
*IGLV3-25*	1	27	1	27					FCGR activation		
*IGLV7-43*	1	48	1	48							
*SYK*	1	2	1	2							
*IGLV3-25*	1	27	1	27					Regulation of actin dynamics for phagocytic cup formation		
*IGLV7-43*	1	48	1	48							
*SYK*	1	2	1	2							
*IGLV3-25*	1	27	1	27					Role of LAT2/NTAL/LAB on calcium mobilization		
*IGLV7-43*	1	48	1	48							
*SYK*	1	2	1	2							
*IGLV3-25*	1	27	1	27					Role of phospholipids in phagocytosis		
*IGLV7-43*	1	48	1	48							
*SYK*	1	2	1	2							
*IGLV3-25*	1	27	1	27					FCGR3A-mediated IL10 synthesis	Leishmania infection	
*IGLV7-43*	1	48	1	48							
*SYK*	1	2	1	2							
*IGLV3-25*	1	27	1	27					FCGR3A-mediated phagocytosis		
*IGLV7-43*	1	48	1	48							
*SYK*	1	2	1	2							
*AMBN*			1	53			1	24	Post-translational protein phosphorylation	Metabolism of proteins	
*CP*			1	34			1	22			
*PENK*			1	29			1	25			
*AMBN*			1	53			1	24	Regulation of Insulin-like Growth Factor (IGF) transport and uptake by Insulin-like Growth Factor Binding Proteins (IGFBPs)		
*CP*			1	34			1	22			
*PENK*			1	29			1	25			
*AOX1*			1	85					Drug metabolism cytochrome p450		KEGG
*CYP2B6*			1	45							
*FMO3*			1	113							
*GSTK1*			1	67							
*OR1N2*	1	41	1	41	1	58	1	58	Olfactory transduction		
*OR2AG2*	1	61	1	61	1	39	1	39			
*OR52B2*	1	54	1	54	1	29	1	29			
*OR52M1*	1	488	1	119	1	287	1	54			
*OR6V1*	2	245	1	22	1	52	1	52			
*OR6C6*	1	52	--	--	1	51	1	51			
*OR6C4*	1	138	1	52	1	32	--	--			
*GUCA1C*	2	937	--	--	2	425	--	--			
*OR10C1*	2	664	--	--	2	388	--	--			
*OR51E1*	1	164	--	--	1	109	--	--			
*OR51G1*	1	1458	--	--	1	835	--	--			
*OR6C70*	1	470	--	--	1	259	--	--			
*CROT*	1	317	1	317	1	160	1	159	Peroxisome		
*GSTK1*	1	67	1	67	1	51	1	51			
*EHHADH*	2	119	1	119	2	58	2	58			
*PEX7*	2	106	1	106	2	47	2	47			
*AMPD1*			1	52			1	25	Purine metabolism		
*ATIC*			1	73			1	47			
*NUDT2*			1	34			1	14			
*PDE11A*			1	115			1	65			
*CYP1A1*			1	62					Retinol metabolism		
*CYP2B6*			1	45							
*DGAT2*			1	116							
*RPE65*			1	52							
*AOX1*			1	85					Tryptophan metabolism		
*CYP1A1*			1	62							
*EHHADH*			2	119							

## Discussion

We performed gene- and pathway-based RVAS using lymphoid cancer cases with a strong family history and early cancer onset. Choosing cases with a strong family history and early onset is expected to enrich for cases with a genetic component to their disease. These families often have members with different lymphoid cancer types and subtypes. We suspect that some genes could predispose to more than one type of lymphoid cancer in such families. Previous rare variant association studies have analysed only non-Hodgkin lymphoid cancers collectively [32]. The genes *EHHADH*, *INTU*, *PEX7*, and *ASXL1 were found to have a significantly higher burden of deleterious variants in cases than controls; variants in these genes* may contribute to risk of lymphoid cancers in specific individuals. However, the variants of interest segregated with lymphoid cancer in other affected members of the same family, in only two families, a variant in *INTU* in family 100 and a variant in *PEX7* in Family 129.

*PEX7* encodes the peroxin 7 protein required for the peroxisomal importation of proteins. To our knowledge, the gene has not been previously implicated in lymphoid neoplasms, but peroxisomal proteins may have a role in the regulation of innate and adaptive immune cells by controlling the synthesis and breakdown of immune bioactive metabolites such as reactive oxygen species and reactive nitrogen species [56, 57]. *Drosophila* Schneider 2 (S2) macrophage-like cells with *PEX7* and *PEX5* knocked down by RNA interference fail to perform phagocytosis, suggesting that compromised peroxisome function within macrophages may possibly reduce the immune system’s ability to eliminate cancer cells [58].

*INTU* is involved in Hedgehog signalling, a pathway activated in various cancers, including basal cell carcinoma, medulloblastoma and hematological malignancies [59, 60].

Variants in *EHHADH* and AXSL1 did not segregate with lymphoid cancer in the families studied, which implies that the variants do not play a role in all affected members of these families, it does not rule out their making a contribution to cancer predisposition in the original case. *EHHADH* encodes a peroxisome enzyme that oxidises medium-chain and large-chain fatty dicarboxylic acids and has delta 3, delta 2-enoyl-CoA isomerase activity [61]. It has been suggested that *EHHADH* affects T-cell function in tumor infiltration, affecting immune surveillance in cancer [62]. The *EHHADH* variant NM_001966.4:c.594_595del is reported in Clinvar as a variant of unknown significance. *ASXL1* plays a role in chromatin remodelling and is a tumor suppressor gene [63]. Several *ASXL1* somatic mutations have been described in CLL [64, 65]. The NM_015338.6:c.1934dup (rs750318549) variant observed in *ASXL1* is a known mutation found in >50% of myeloid malignancies [66]. Germline variants in *ASXL1* must be interpreted with caution, particularly in older individuals, as variants in this gene observed in blood DNA could be of somatic in nature [67, 68]. A candidate gene-based study by Hamadou *et al*. (2016) identified a c.1205 G>A germline missense substitution in *ASXL1* in two sisters, one with large B-cell lymphoma and the other with mucosa-associated lymphoid tissue lymphoma [69]. The c.1205 G>A missense variant is different than the one identified here, but the affected family members in Hamadou *et al*. (2016) and our study have different lymphoid cancer subtypes; this may imply a role for different *ASXL1* variants in cases with different lymphoid cancer subtypes.

Pathway-based burden analysis uses pathways from KEGG, Reactome, and Biocarta. These databases are generated mainly as an independent effort by experts, with each database having specific criteria for annotation and speed of curation; there are redundant pathways amongst the databases which would make the multiple testing correction overly conservative. The Reactome pathways of innate and adaptive immune systems showed association with lymphoid cancers. Previous studies have demonstrated the involvement of variants in immune system related genes in different subtypes of lymphoid cancer [70–72]. These studies, combined with our results, support that genetic variation in immune-related genes plays a role in the development of lymphoid neoplasms. *SYK*, *IGLV3*-*25*, and *IGLV7-43* are involved in the innate and adaptive immune pathways defined in Reactome. SYK is a spleen tyrosine kinase activated by binding to the phosphorylated Immunoreceptor tyrosine- based activation motif, triggering a downstream signalling cascade regulated by its tyrosine phosphorylation [73]. This gene plays a role in cell adhesion, innate pathogen recognition, platelet activation, and vascular development [74]. *SKY* amplification has been identified as a genetic alteration in primary BCR-type DLBCL tumours [75]. *IGLV3-25* and *IGLV7-43* are immunoglobin light chain variable regions involved in antigen recognition. Cells that express a point mutation that changes amino acid position 110 from glycine to arginine in *IGLV3-21* trigger autonomous B-cell receptor (BCR) signalling by BCR-BCR interaction and confer aggressive behaviour in CLL [76]. The pattern of somatic mutation in sporadic CLL for IGLV3-21*04 and R110 is similar to that of familial cases of CLL [77]. *BLK*, a B-lymphoid tyrosine kinase involved in the adaptive pathway, is a proto-oncogene and a member of the SRC family kinases involved in B-cell development [78]. This gene has been identified in a family of two members affected with Hodgkin lymphoma [79]. The case with the variant in the proto-oncogene *BLK* also had a variation in *AXSL1*, a tumour suppressor gene. *GRAP2* is a tumour suppressor gene involved in T cell signalling, development and regulate NF-AT activation [80]. *GRAP2* has been implicated in extranodal NK/T cell lymphoma, a rare and aggressive subtype of T cell or NK lineage extranodal lymphoma [81]. *GARP2* is a specific marker of T lineage differentiation; it was interesting to observe a variant in this gene in a case affected with CLL, suggesting a role of *GRAP2* in B cell lymphoma as well. These genes would not have been detected if pathway-based burden analysis had not been performed.

The KEGG pathway that was associated, for both ≤1% and ≤0.1% allele frequencies and both control groups, was olfactory transduction; this pathway had the highest number of genes, 12, with rare variants. Although there is no previously known involvement of olfactory genes in lymphoid cancers, these genes have been studied in other neoplasms such as prostate cancer; for instance the activation of *OR51E2* facilitates the cellular transformation of the disease into a more aggressive form [82]. Olfactory gene OR2B6 is expressed in over 80% of breast carcinoma tissues and its expression has been suggested as a potential biomarker in breast carcinoma [83]. It is also not implausible that this pathway could influence the sense of smell of individuals and contribute to avoidance of exposure to some carcinogens, thus affecting cancer risk. Olfactory receptor genes are a large gene family with over 900 genes and pseudogenes [84], which can complicate the mapping of sequenced reads and lead to erroneous genotypes and potentially spurious association. Though all variants of interest in this study were individually verified by examining them in IGV, this may not be sufficient to eliminate this type of error.

This study has three strengths: first, it uses cases with early onset and a strong family history of lymphoid cancers; it thus enriches for cases that are likely to have a stronger genetic component to their disease. Second, it enables investigation of variants of interest in other affected family members. Third, we assess rare variant associations at both the gene- and pathway-level, improving the possibility of making meaningful inferences regarding the etiology of lymphoid cancers, which might not have been possible by analysing individual genes.

This work also has several limitations. The use of public control datasets can give rise to issues due to differences in population structure and sequencing methods, including differences in depth of sequencing and in data processing. Some of these potential issues were limited; however, by restricting analyses to variants with ≥10x coverage in both controls and cases, as suggested by Guo *et al*. [34].

A second limitation is calculation of the inflation factor lambda. The inflation factor lambda reflects the inflation in the test statistic due to ancestry; not correcting for this factor can lead to false associations. TRAPD calculates the observed and expected -log_10_(p values) of lambda at the 95^th^ percentile with the highest expected -log_10_ (p value) for genes with p-value 1.0; however, in our study we only included genes with rare variants present in the cases, making it unfeasible to use the TRAPD inflation factor calculation. Instead, the inflation factor lambda_0.5–0.95_ was calculated as proposed by Chen *et al*., 2021 for burden analysis using public datasets as controls; this method excludes inflated value at the extremes of the plot [53]. The inflation factor in some instances was >1, indicating potential confounding by genetic background; this was offset by correcting for the inflation factor by multiplying it with the p-value. Yang *et al*. (2011) showed substantial inflation in the context of polygenic inheritance, even when data was controlled for population structure and technical artifacts [85]. The inflation >1 observed in lymphoid neoplasms might also be due to polygenic inheritance.

The success of rare variant association analyses depends on various factors, two of which are statistical power and a suitable method for the association study. RVAS suffers from low statistical power due to the rarity of the variants being tested. Attempts to improve statistical power in RVAS are made by increasing the sample sizes or collapsing rare variants into gene annotation categories and biological functions. In this RVAS of lymphoid cancers, we considered genes with two or more variants, as done by others [86, 87], and also carried out pathway-based analyses. These analyses reduce the number of tests, thereby preserving power. For disease-causing genes in lymphoid cancers, we expect that a majority of rare causal variants will increase disease risk. Given that burden analysis assumes that all the variants influence the phenotype in the same direction [29], the selection of this test seems appropriate for our analyses.

## Conclusion

RVAS was performed using lymphoid cancer cases with a strong family history and early disease onset. Cases with early–onset and family history are more likely to have a genetic basis than later onset cases. Publicly available data sets with large sample sizes were used as controls to maximize statistical power of burden analysis. Nevertheless, the case sample size was small and therefore statistical power was limited; large studies are necessary to substantiate these findings. We identified putative pathogenic germline variants in four genes, and biological pathways related to immune systems, as possible contributors to lymphoid cancer risk. Stop-gain variant NM_015693.4:c.581G>A in *INTU* in the Hedgehog signalling pathway and stop-gain variant NM_000288.4:c.875T>A in *PEX7* in peroxisomal pathway each segregated with lymphoid cancers in families, suggesting that these variants in these genes may be related to risk of lymphoid cancer.

## Supporting information

S1 Fig Principal component analysis of 39 lymphoid cancer cases performed using KING (version 2.2.7) with the 1000 Genome dataset as reference. Thirty-eight cases with European ethnicity were selected for the rare variant association study while one case of non-European ancestry was removed.
(TIFF)

S1 Table The inflation factor (lambda_0.5–0.95_) for the association tests of lymphoid cancer cases vs. GnomAD exome and ExAC controls, at allele frequency ≤0.1% and ≤1%. (XLSX)

S2 Table Statistical power calculation for more than 2 variants analysis with lymphoid cancer cases and GnomAD or ExAC controls, for allele frequencies ≤1% and ≤0.1%. (XLSX)

S3 Table Genes with two or more variants and count of variants in lymphoid cancer cases and GnomAD or ExAC controls, for allele frequencies ≤1% and ≤0.1%. (XLSX)

S4 Table Pathways and count of variants in lymphoid cancer cases and GnomAD or ExAC controls, for allele frequencies ≤1% and ≤0.1%. (XLSX)
