# Supplementary figures and images for: Gene and pathway based burden analyses in familial lymphoid cancer cases: Rare variants in immune pathway genes
Source: PLoS One. 2023 Jun 28;18(6):e0287602. doi: 10.1371/journal.pone.0287602 (PMC10306212; doi:10.1371/journal.pone.0287602)

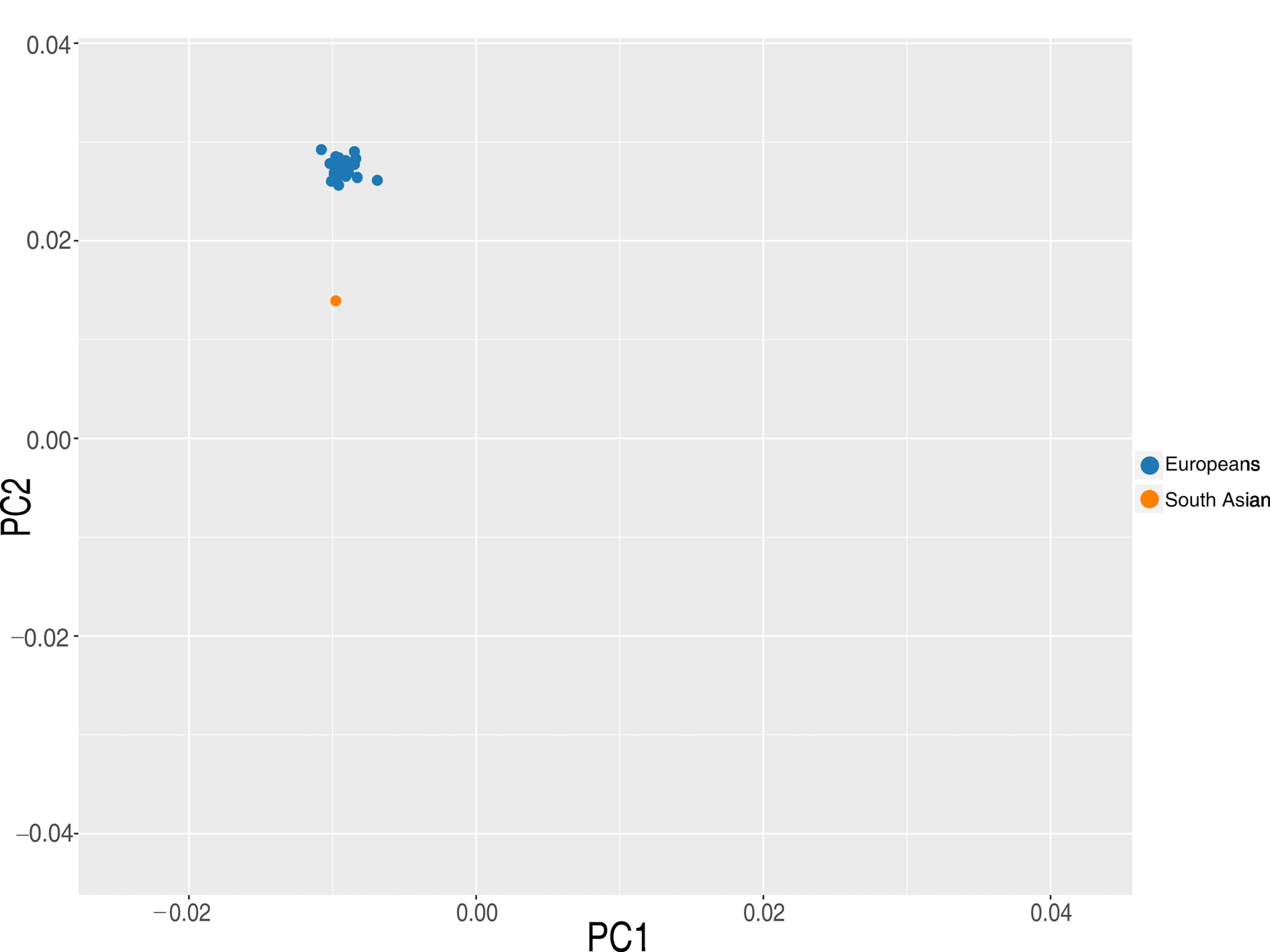

Supplement: S1 Fig — Thirty-eight cases with European ethnicity were selected for the rare variant association study while one case of non-European ancestry was removed. (TIFF) [file pone.0287602.s001.tiff]
